# Seasonal patterns and spatial variation of *Borrelia burgdorferi* (*sensu lato*) infections in *Ixodes ricinus* in the Netherlands

**DOI:** 10.1186/s13071-021-04607-7

**Published:** 2021-02-24

**Authors:** Nienke Hartemink, Arnold J. H. van Vliet, Gerrit Gort, Fedor Gassner, Frans Jacobs, Manoj Fonville, Willem Takken, Hein Sprong

**Affiliations:** 1 Biometris, Wageningen University and Research Centre, Wageningen, The Netherlands; 2 Environmental Systems Analysis Group, Wageningen University, Wageningen, The Netherlands; 3 Gassner Biological Risk Consultancy, Houten, The Netherlands; 4 Centre for Monitoring of Vectors, National Reference Centre, Netherlands Food and Consumer Product Safety Authority, Wageningen, The Netherlands; 5 Centre for Infectious Disease Control, National Institute for Public Health and the Environment, Bilthoven, The Netherlands; 6 Laboratory of Entomology, Wageningen University and Research Centre, Wageningen, The Netherlands

**Keywords:** Lyme borreliosis, Acarological risk, Prevalence, Seasonality

## Abstract

**Background:**

The incidence of Lyme borreliosis varies over time and space through as yet incompletely understood mechanisms. In Europe, Lyme borreliosis is caused by infection with a *Borrelia burgdorferi* (*s.l.*) genospecies, which is primarily transmitted by a bite of *Ixodes ricinus* nymphs. The aim of this study was to investigate the spatial and temporal variation in nymphal infection prevalence of *B. burgdorferi* (*s.l.*) (NIP), density of questing nymphs (DON) and the resulting density of infected nymphs (DIN).

**Methods:**

We investigated the infection rates in* I. ricinus* nymphs that were collected monthly between 2009 and 2016 in 12 locations in the Netherlands. Using generalized linear mixed models, we explored how the NIP, DON and DIN varied during the seasons, between years and between locations. We also determined the genospecies of the *Borrelia* infections and investigated whether the genospecies composition differed between locations.

**Results:**

The overall NIP was 14.7%. A seasonal pattern in infection prevalence was observed, with higher estimated prevalences in the summer than in the spring and autumn. This, combined with higher nymphal densities in summer, resulted in a pronounced summer peak in the estimated DIN. Over the 7.5-year study period, a significant decrease in infection prevalence was found, as well as a significant increase in nymphal density. These two effects appear to cancel each other out; the density of infected nymphs, which is the product of NIP × DON, showed no significant trend over years. Mean infection prevalence (NIP, averaged over all years and all months) varied considerably between locations, ranging from 5 to 26%. *Borrelia* genospecies composition differed between locations: in some locations almost all infections consisted of *B. afzelii*, whereas other locations had more diverse genospecies compositions.

**Conclusion:**

In the Netherlands, the summer peak in DIN is a result of peaks in both NIP and DON. No significant trend in DIN was observed over the years of the study, and variations in DIN between locations were mostly a result of the variation in DON. There were considerable differences in acarological risk between areas in terms of infection prevalence and densities of ticks as well as in *Borrelia* genospecies composition.
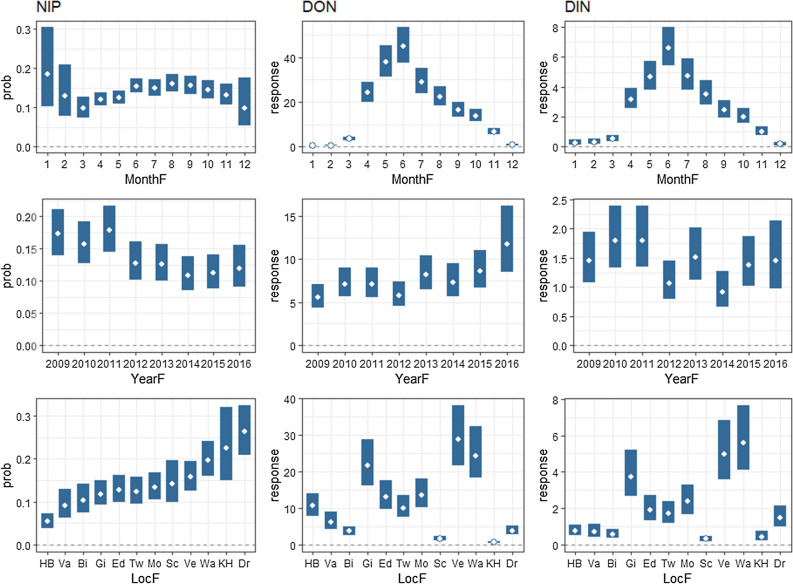

## Introduction

Lyme disease, or Lyme borreliosis, is the most prevalent tick-borne infection of humans in the northern hemisphere. In western Europe, its causative bacterial agent, *Borrelia burgdorferi* (*s.l.*) (referred to as *Borrelia* hereafter), and its vector, the caster bean tick *Ixodes ricinus*, are widespread. A Europe-wide meta-analysis on *Borrelia* prevalence found an average prevalence of 5.9% in* I. ricinus* nymphs in western Europe [39]. In the Netherlands, both *Ixodes ricinus* and the *Borrelia* spirochaete have been found in various habitats across the country and to vary considerably in space [6, 13, 43]. A study conducted between 2000 and 2004 found significant differences between four study habitats (heather, forest, city park and dunes) and between years, with *Borrelia* prevalence ranging from 0.8 to 11.5% [43]. Analysis of the first 1.5 and 5 years of the current study yielded estimates for the mean prevalence per location of between 0 and 29% and 7 to 26%, respectively [13, 40].

In the last few decades, the number of reported tick bites and the incidence of erythema migrans (an early sign of Lyme borreliosis) reported by general practitioners in the Netherlands have increased sharply [18, 19] (see also Tekenradar 2020 [42]). This could partly be a result of increasing awareness among general practitioners and the general public, but there are also indications that the number of ticks is increasing as well [37]. The risk of acquiring Lyme disease depends on many different factors, of which the most important are the abundance of questing *I. ricinus* ticks infected with *Borrelia* [24] and the level of human exposure to ticks [21, 44]. Therefore, a proper understanding of the spatial and temporal variation in risk of being bitten by an infected tick is necessary for effectively informing the public, so that people know when and where preventive measures should be implemented. Examples of preventive measures include checking for tick bites after outdoor activities, wearing tick-repelling clothes and/or avoiding certain areas at certain times of the year altogether.

Since *I. ricinus* nymphs are responsible for most bites on humans, the risk of acquiring an infected tick bite during a given activity is largely determined by the density of infected nymphs (DIN) [8, 10, 24]. The DIN is therefore also referred to as the acarological risk of exposure to tick-borne pathogens [10, 27, 40]. The DIN is determined by the density of questing nymphs (DON) and the nymphal infection prevalence (NIP). The DON can be considered to be a proxy for the probability of acquiring a tick bite, whereas the NIP can be regarded as an estimate of the probability that the bite is from an infected tick.

Within the framework of the Dutch phenological citizen science programme Nature’s Calendar, nymphs were collected monthly by well-trained volunteers at 12 locations throughout the Netherlands for a period of 10 years [13, 15, 40]. For the period 2009–2016, captured nymphs were screened for the presence of *B. burgdorferi* (*s.l.*) using quantitative real-time PCR (qPCR). This longitudinal dataset, consisting of 7.5 years of prevalence data, allowed us to determine the spatial and temporal variation in *Borrelia* NIP, the specific *B. burgdorferi* genospecies, the DON and the DIN.

We determined the *B. burgdorferi* genospecies for the infected nymphs when possible. Different genospecies have been associated with different manifestations of Lyme borreliosis; for example *Borrelia afzelii* is mostly associated to erythema migrans, whereas *B. garinii* has been associated with Lyme neuroborreliosis [7, 38]. Since the genospecies are also associated with different reservoir hosts (*B. afzelii* with small mammals and *B. garinii* and *B. valaisiana* with bird species)[23] and locations are likely to differ in their vertebrate species composition, we investigated whether *Borrelia* genospecies composition differed between locations.

## Materials and methods

### Tick collection

**Fig. 1:**
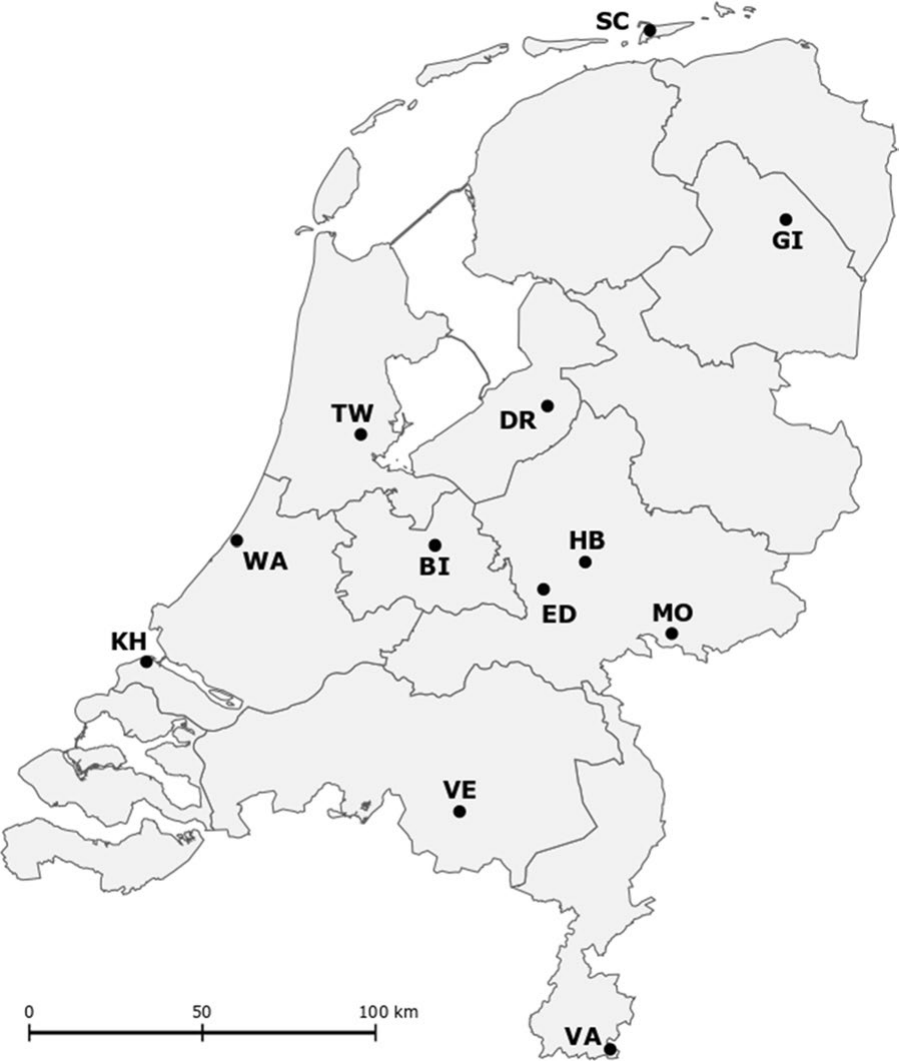
Overview of the sampling sites in the Netherlands. *BI* Bilthoven, *DR* Dronten, *ED* Ede, *GI* Gieten, *HB* Hoog Baarlo, *KH* Kwade Hoek, *MO* Montferland, *SC* Schiermonnikoog, *TW* Twiske, *VA* Vaals, *VE* Veldhoven, *WA* Wassenaar

Nymphs were collected by well-trained volunteers at 12 locations in the Netherlands. The sampling was part of the above-mentioned Nature’s Calendar project and has been described in detail elsewhere [13, 15, 40]. Details on the 12 sampling locations can be found in Appendix A, and a map is shown in Fig. 1. Each location was sampled once a month by dragging a white cotton cloth (1 m^2^) along two marked 100-m-long transects; thus, the total sampled area per location was 200 m^2^ per session. The cloth was inspected for ticks at intervals of 25 m. All larvae, nymphs and adult ticks that had attached to the cloth were counted and collected at each interval. Upon arrival in the laboratory, the ticks were identified by an experienced technician using morphological keys as described in [1] and [17]. All captured ticks were *I. ricinus*. In this analysis, we included nymphs collected between January 2009 and June 2016, as nymphs collected before January 2009 were screened for *Borrelia* infections with a different method. In total, 26,658 nymphs have collected since January 2009. The maximum number of nymphs to be tested per sampling session was set at 30 (or 60 in later years); in total, 14,910 nymphs were tested.

### Testing for *Borrelia*

Only nymphal ticks were examined for the presence of *Borrelia* DNA. If on any sampling day and site fewer than 30 nymphs were collected, all ticks were examined; if more than 30 nymphs were found, a random sample of approximately 30 specimens collected at that site was selected for *Borrelia* analysis. For all samples, DNA was extracted by alkaline hydrolysis [14]. DNA extracts were stored at − 20 °C until further use. For the detection of *Borrelia* DNA, a duplex qPCR was used, based on the detection of fragments of the outer surface protein A (ospA) and flagellin genes [16]. DNA from the samples that were qPCR-positive were amplified by conventional PCR that targeted the 5S–23S ribosomal RNA intergenic spacer region of *B. burgdorferi* as described [5]. In short, we used the forward primer B5Sborseq (5′-GAGTTCGCGGGAGAGTAGGTTATTGCC-3′) and the reverse primer 23Sborseq (5′-TCAGGGTACTTAGATGGTTCACTTCC-3′). To check whether a product was obtained from the PCR, 8-μl aliquots of the PCR mixture were electrophoresed in a 1.5% agarose gel containing an electrophoretic color marker (SYBR™ Gold Nucleic Acid Gel Stain; Invitrogen™, Carlsbad, CA, USA). If the PCR was successful and a clear band was visible on the gel, the DNA was cleaned with ExoSAP-IT™ PCR Product Cleanup Reagent (Applied Biosystems™, Foster City, CA, USA) and sent for sequencing at the commercial laboratory BaseClear BV (Leiden, the Netherlands). The chromatographs of the sequences were visually inspected and the primers sites trimmed in Bionumerics version 7.4 (Applied Maths, Sint-Martens-Latem, Belgium). These sequences were used to identify the *B. burgdorferi* (*s.l.*) genospecies by comparison to sequences of known genospecies from GenBank. The cluster analyses and genospecies determination were also performed in Bionumerics version 7.4 (Applied Maths) exactly as described previously [5].

### Statistical analysis of the NIP, DON and DIN

For the analysis of the NIP, DON and DIN, all data were used, except for the observations from Vaals between December 2013 and June 2016, where the sampling effort had changed due to one of the transects not being sampled in this period. As a result, 465 nymphs (359 were tested of which 44 were positive for *Borrelia*) were omitted, leaving 26,193 nymphs (14,551 were tested, of which 2151 were positive for *Borrelia*) for the analysis. For each sampling session (that is, each specific combination of location, month and year), the NIP was calculated as the number of *Borrelia*-positive nymphs divided by the number of tested nymphs. The DON is the total number of nymphs collected at a session, and the DIN is calculated as the NIP multiplied by the DON (which for sessions in which all nymphs were tested is the same as the number of collected nymphs that were *Borrelia* positive). Both DON and DIN are expressed as the number of ticks per 200 m^2^ of sampled area. All analyses were performed in R version 3.2.5 [30].

We used generalized linear mixed models (GLMMs) to analyse how the NIP, DON and DIN depend on the year, month and location using different models for each of these tick parameters. GLMMs were selected because these models can handle non-normally distributed data, such as count data, as well as (multiple) random effects. We used the glmmTMB package [2], which handles beta-binomial and negative binomial distributions as well as crossed and nested random effects. For the NIP we assumed a beta-binomial distribution, and a logit link function. For the DON and DIN, we used a negative binomial distribution with a log link.

We included the variables Location, Year and Month as fixed effects. Locations within Year were included as random effect (1 | YearF:LocF) to account for the fact that observations from the same year–location combination are not independent. With the drop1() function, we dropped single terms into the model one by one to see whether the variables Location, Year and Month had any predictive power in the model. Estimated marginal means were calculated for each month, year and location, using the emmeans package [22]. Estimated marginal means should be interpreted as predicted averages over all factors (based upon the fitted model), so the estimated marginal mean NIP for a specific month was averaged over all years and all locations. We also tested for (linear) trends over the years by running the GLMM models with year as numerical fixed factor.

### Analysis of genospecies

For the analysis of the variation in genospecies composition, data from all locations and all years were used. The analysis was performed on the 1298 infected nymphs from which the genospecies was determined. The distribution over the different genospecies was plotted in pie charts, both for all locations together and for separate locations.

## Results

### NIP, DON and DIN

**Fig. 2:**
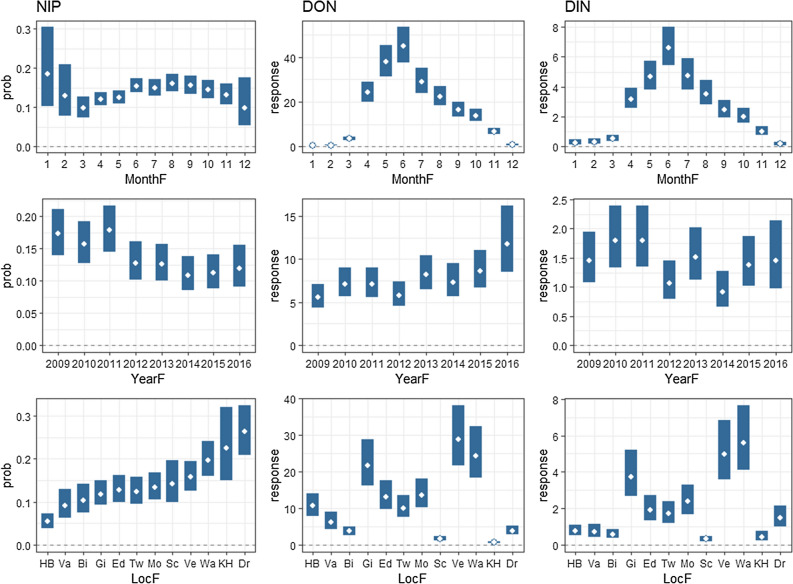
Estimated marginal means for nymphal infection prevalence (*NIP*), density of nymphs (*DON*) and density of infected nymphs (*DIN*) for the different months, years and locations of the study. The* x-axes* of the graphs for the locations (lower three panels) were sorted based on the prevalence values. Code for the locations is given in caption to Fig. 1

Of the 14,910 tested nymphs, 2195 were found to be positive for *Borrelia burgdorferi* (*s.l.*) Therefore, the overall prevalence in this study was 14.7%. Dropping the single fixed terms into the model one by one using the drop1() function yielded significant effects (*α* = 0.05) for Year, Location and Month for each of the three outcome variables, indicating that there is spatial and temporal variation in NIP, DON and DIN. The estimated marginal means for the NIP, DON and DIN are shown for each of the months, years and locations in Fig 2. We present the results for seasonal variation (between-month variation), temporal variation over years and spatial variation (between locations) separately. The raw data for NIP, DON and DIN (calculated as the product of NIP × DON) are presented per year and location (Appendix B) and per season and location (Appendix C).

### Seasonal variation

The NIP shows a seasonal pattern, with a gradual increase in prevalence from March to August and a decrease after August. In a pairwise comparison, the estimated marginal means for March (9.7%, 95% confidence interval [CI] 7.3–12.8%) and August (16.1%; 95% CI 14.0–18.5 %) differed significantly (*p* = 0.032); all other comparisons yielded* p* values > 0.05 (although for March–June, March–September, April–June and April–August they were < 0.10). In the winter months, prevalence seems to be higher than in spring, but these estimates are based on a very limited number of nymphs captured (and tested) and they are therefore less reliable. The DON shows a clear unimodal pattern, with a steep increase between March and June, and a decrease from June to winter. The DIN shows a very similar pattern, but due to the NIP peak being slightly later than the DON peak, the peak in DIN is more symmetrical than that in the DON; that is, the DON is higher in May than in August, but since NIP peaks in August, the DIN values in May and August become similar.

### Temporal variation over years

A significant decreasing trend in NIP was observed over the 7.5 years of the study period (*p* < 0.001). The overall estimated odds ratio for year is 0.92, which means that the odds of the tick being infected with *Borrelia* in 1 year is 0.92-fold the odds in the previous year. A significant increasing trend in the DON over the study years was also observed (*p* < 0.01), with an estimated ratio between the DON in 1 year and the preceding year of 1.07. The DIN, which is the resulting variable of the latter two, does not show a trend (*p* = 0.10). We checked if this pattern was the same for all individual locations. For several locations, the model did not converge, but for the locations where we had estimates, the trends are similar to the overall trend, with the exception of Ede, Dronten and Hoog Baarlo, where the DIN shows a significant negative trend (Table 1; see Appendix E for more detailed results).

**Table 1:** Estimated linear trends over time for the NIP, DON and DIN for each location and for all locations combined

Sampling sites	NIP^a^		DON^b^		DIN^b^	
	Odds ratio	*p* value	Ratio	*p* value	Ratio	*p* value
Bilthoven	0.82	< 0.01	1.18	0.01	0.95	0.49
Dronten	0.93	0.32	0.83	0.02	0.80	0.01
Ede	0.84	< 0.01	1.02	0.76	0.80	< 0.001
Gieten	0.96	0.39	1.11	0.07	1.05	0.43
Hoog Baarlo	0.81	< 0.01	1.18	< 0.01	0.86	0.02
Kwade Hoek	1.03	0.13	0.99	0.94	1.13	0.56
Montferland	0.90	0.05	1.04	0.37	0.92	0.22
Schiermonnikoog	1.01	0.89	1.00	0.99	0.94	0.47
Twiske	0.83	< 0.001	1.10	0.04	0.91	0.21
Vaals	NA^c^	NA^c^	0.92	0.28	1.03	0.73
Veldhoven	0.99	0.93	1.27	< 0.001	1.11	0.23
Wassenaar	0.95	0.04	1.10	0.02	1.08	0.09
All locations	0.92	< 0.001	1.07	< 0.01	0.96	0.10

DIN, Density of infected nymphs; DON, density of questing nymphs; NIP, nymphal infection prevalence

^a^Expressed as the odds ratio, that is, the ratio between the odds of ticks being infected in two consecutive years

^b^Expressed as the ratios between the rates of (infected) ticks in consecutive years

^c^NA indicates that the model-fitting algorithm did not converge

### Spatial variation

The estimated marginal means (averaged over year and months) of NIP, DON and DIN vary substantially between locations (see three lower panels of Fig. 2). The estimated mean NIP varied between 5% (95% CI 4.0–7.3%) in Hoog Baarlo and 26% (95% CI 21.0–32.5%) in Dronten. The results of a pairwise comparison of the NIP values between the locations show that Hoog Baarlo has a significantly lower NIP than most locations and Dronten has a significantly higher NIP (Appendix F). There are also considerable differences in DON between the locations. The DIN values show a very similar pattern to the DON values; locations with high DON values (Gieten, Veldhoven and Wassenaar) also have high DIN values and,* vice versa*, locations with low DON values have low DIN values (Schiermonnikoog, Bilthoven and Vaals); the same applies to intermediate DON/DIN values (Ede, Montferland and Twiske). In cases of low (Hoog Baarlo) or high values of NIP (Kwade Hoek and Dronten), the DIN is lower and higher, respectively, than would be predicted just from the DON. We quantified how much of the variation in DIN between locations was attributable to variation in NIP and DON, respectively, by comparing the effect sizes. The effect size of (standardized) DON was approximately twice as high as the effect size of (standardized) NIP, which indicates that the value of the DIN is to a large extent determined by the DON.

### Genospecies

Of the 2195 *Borrelia*-infected nymphs, genospecies was determined for 1298 infected nymphs. For the remaining nymphs, either no attempt was made to determine the genospecies (505 infected nymphs collected between January 2010 and June 2011) or the genospecies could not be determined (392 nymphs).

**Fig. 3:**
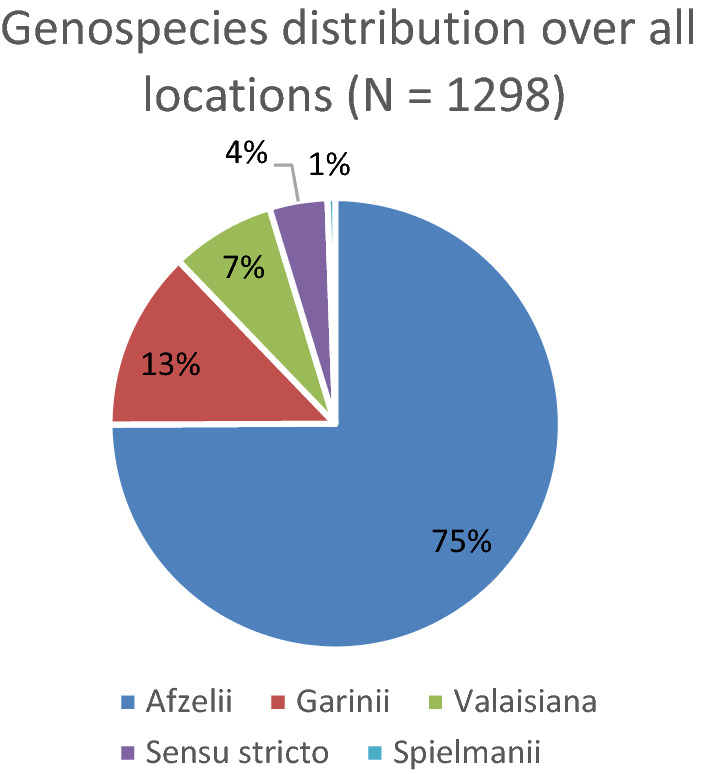
Distribution of* Borrelia* genospecies (within the 1298 infected nymphs for which the genospecies could be determined)

Of of the 1298 nymphs for which the genospecies of the *Borrelia* was determined, the large majority were found to be infected with *B. afzelii* (972 nymphs, 74.9%) (see Fig. 3). The other genospecies were *B. garinii* (167 nymphs, 12.9%), *B. valaisiana* (97 nymphs, 7.5%), *Borrelia* (*s.s.*) (54 nymphs, 4.2%), and *B. spielmanii* (only 7 nymphs, 0.5%). *Borrelia turdi* was found only once (0.05%) (not shown in Fig. 3). In four locations, > 90% of the tick infections were of the genotype *B. afzelii* (Kwade Hoek, Twiske, Veldhoven and Wassenaar) (Appendix D). In Vaals, the genospecies composition was highly diverse, with 33% *B. afzelii*, 28% *Borrelia *(*s.s.*), 25% *B. garinii*, 11% *B. valaisiana*, 2% *B. spielmanii* and 1% *B. turdi*. In the other seven sampling locations, *B. afzelii* was dominant (between 55 and 70%), but the combined percentages of *B. garinii*, *B. valaisiana*, and in some cases *B. burgdorferi* (*s.s.*) comprised at least 30% of the genospecies present. Temporal trends were not assessed because the number of infections with specific genospecies per location was too low.

## Discussion

In this study, based on monthly data collected from 12 sampling locations throughout the Netherlands over a 7.5-year period, 2195 of the 14,910 nymphs assayed were found to be positive for *B. burgdorferi* (*s.l.*). The overall NIP (i.e., the overall percentage of all tested nymphs regardless of year, month and location) was thus 14.7%. We found marked differences in prevalence through the season, over time and between locations.

The 14.7% prevalence in nymphs is in line with previous findings from a meta-analysis for *B. burgdorferi* (*s.s.*) prevalence in questing nymphs in Europe, where the prevalence was estimated at 12.3, 14.6 or 15.4%, depending on whether the detection was done with PCR, nested PCR or qPCR, respectively [39]. Our findings are in line with reported prevalences in the neighbouring country Belgium, where studies reported estimates for the prevalence of *Borrelia* in nymphs of 15.6% [34] and in ticks of 12% [20]. However, it is higher than the mean *Borrelia* infection prevalence of 5.9% that was reported for nymphs in western Europe (defined here as Belgium, France, Luxembourg, and the Netherlands) in the meta-analysis by Strnad and colleagues [39].

Our study revealed a striking seasonal pattern in prevalence, with the NIP steadily increasing between March and August, and decreasing afterwards. In the winter months, the prevalence also appears to be higher as well, but due to low numbers of captured nymphs, these estimates are very uncertain. Information on the seasonal trends in other locations is scarce; studies on the seasonality of questing tick activity often present a single overall estimate for the *Borrelia* infection prevalence (for example, see [9, 28, 36]. Our finding of an increase between spring and summer is in concordance with findings in southern Germany, where nymphal infection prevalence was found to rise from 9.3% in the spring to 16.1% in the summer [11]. In Sweden and Norway, different patterns have been observed, with higher prevalences in late spring and early summer, compared to late summer and autumn [25, 41]. A study in France on 461 questing nymphs found no differences throughout the year [29], but that may be linked to the limited study size. A study in southwestern Slovakia reported that the prevalence of tick-borne pathogens followed the same pattern as that of tick questing activity, namely a bimodal seasonal pattern with a peak in April and May and another one in July [4]. However, this latter study reports the relative prevalence, which is the proportion of positive ticks for the given pathogen during a certain month divided by the total number of collected ticks over the whole period, not the monthly prevalence as used in our study. A study in Luxembourg reported a bimodal seasonal activity beginning with high numbers of *Borrelia* infections in ticks in May and a second peak in September. This study, conducted between May and October, was based on 157 infected nymphs of a total of 1394 nymphs and adult ticks [32]. Our study, which reports year-round monthly values for the NIP during a period of 7.5 years and which is based on almost 15,000 tested nymphs (of which 2195 tested positive for *Borrelia*) provides a unique opportunity to study the seasonality of *Borrelia* prevalence in questing *Ixodes ricinus* nymphs in such detail. As possible mechanisms underlying the seasonal trend in infection prevalence in questing nymphs, we speculate that seasonality in host availability (e.g. rodent populations are strongly seasonal, with lower numbers in winter) or seasonality in the *Borrelia* prevalence in hosts may play a role. The latter would in turn be an effect of seasonality in infected tick bites and host immunity, so cause and effect cannot easily be distinguished. It could also be that infection with *Borrelia* affects the activity levels [12], so that infected nymphs may quest more in winter (which would explain the non-significant higher infection prevalence in winter), possibly leading to depletion of their energy and lower survival through the winter. More information on infection prevalences in host species and on survival in infected and non-infected ticks is needed before we can draw any conclusions on which mechanisms play a role here.

The overall temporal trend over the years for *Borrelia* prevalence in our 7.5-year study period was negative; the NIP decreased slightly between 2009 and 2016 in these 12 locations. By contrast, the density of questing nymphs (DON) increased over the same period. The density of infected nymphs (DIN), which is the product of NIP × DON per session, showed no significant overall trend over the years due to the decrease in NIP and the increase in DON cancelling each other out. In three specific locations, Ede, Hoog Baarlo and Dronten, the DIN decreased significantly. In none of the study locations was a significant increase in DIN observed over the study period. Okeyo et al. [26] studied NIP in the summer period in Latvia and reported a decrease in NIP over the period 1999–2010 (note that the study period differs from the period in our study).

We argue that both NIP and DIN are important measures of risk: NIP is a proxy for the probability that, given a tick bite, one gets infected, whereas the DIN represents a proxy for the probability to get an infected tick bite. A concrete example is as follows: at the transects in Dronten, the chance of getting an infectious tick bite was comparatively low, but once you do get bitten there, the probability of acquiring an infection was high. Other aspects of risk, such as accessibility of the area, contact chance between visitors and tick vantage points, the awareness of the public, among others were not considered in this analysis, but will most certainly also play a role in determining risk profiles.

We also assessed what contributes most to the variation in the DIN: variation in the DON or variation in the NIP. We found that the DIN is mostly determined by the DON, especially when examining variation throughout the season: the reason that the DIN shows a clear seasonal peak is mostly a result of the seasonal peak in DON. Differences in DIN between locations are also mostly a result of differences in DON. This finding is in line with that of Tälleklint and Jaenson [41], who suggested that within a certain range of nymphal densities, it may be possible to assess the density of *Borrelia*-infected *I. ricinus* nymphs without examining nymphs for the presence of *B. burgdorferi * (*s.l.*) [41].

Spatial variation in NIP was substantial during our study period, with estimated prevalence ranging between 5% (in Hoog Baarlo) and 26% (in Dronten). This spatial variation did not coincide with differences in general landscape/habitat characteristics; of the five locations with similar forest type, that is mixed forest with birch and oak with rich understorey (Ede, Hoog Baarlo, Gieten, Montferland and Veldhoven), NIP values at Hoog Baarlo were significantly lower than those at the other locations. Hoog Baarlo, the location with the lowest prevalence among all sampling locations, is also the only location with a population of red deer. Although we cannot draw strong conclusions based on a single location, this finding is in line with reports from previous studies. A study along the West coast of Norway found lower prevalences of *B. burgdorferi* (*s.l.*) in questing *I. ricinus* nymphs in areas of high red deer density [25]. The findings of another study in Norway, on Norwegian islands, also suggests that high abundances of roe deer and red deer may reduce the infection rate of *B. burgdorferi* (*s.l.*) in host-seeking *I. ricinus* [33].

The locations with the highest prevalences were also diverse in terms of vegetation type: the site in Wassenaar is a pine forest near the dunes, whereas Veldhoven is an inland mixed oak–birch forest, and Dronten is situated on reclaimed land and has a mixed of willow and oak in one transect and beech, common alder and sycamore maple in the other. This shows that high prevalences of *Borrelia* can be sustained in several different habitat types.

In all locations, *B. afzelii* was the most prevalent genospecies. Overall, 75% of the infected nymphs was infected with this genospecies. In four locations, *B. afzelii* comprised > 90% of the infections. In seven locations, *B. afzelii* was the dominant genospecies (55–70%), but *B. valaisiana*, *B. garinii* and sometimes *B. burgdorferi* (*s.s.*) also comprised at least 30% genospecies present. In Vaals, the genospecies distribution was more even, with almost equal contributions from *B. afzelii*, *B. burgdorferi* (*s.s.*) and *B. garinii*. The genospecies composition is likely to be a result of the local composition of the host animals. Unfortunately, these have not been recorded in this study. Compared to other studies, our study reports a higher percentage of infections with *B. afzelii*. In several European studies, *B. afzelii* has been reported to be the most prevalent genospecies, but often the *B. afzelii* infections comprised a smaller fraction of the total. A recent meta-analysis reported 45% of infected questing ticks to be infected with *B. afzelii* in western Europe, and percentages of 29% for *B. garinii*, 17% for *B. valaisiana* and 6% for *B. burgdorferi *(*s.s.*) [39]. Another meta-analysis, reporting on studies conducted between 1984 and 2003, found 46% of the reported infections in ticks in the Netherlands, Belgium and northern France to be with *B. afzelii*, 34% with *B. garinii*, 33% with *B. valaisiana* and 19% with *B. burgdorferi* (*s.s.*) [31]*.* In a study in Luxembourg, 33% (*n *= 52) of the infected ticks were infected with *B. afzelii*, 30% with *B. garinii*, 19% with *B. valaisiana,* 15% with *B. burgdorferi *(*s.s.*), 2.5% with *B. spielmanii* and 0.6% with *B. lusitaniae* [32]. In a study in Valais, Switzerland, the percentage of *Borrelia*-positive nymphs infected with* B. afzelii* was 40%, followed by *B. garinii* (22%), *B. valaisiana* (12%) and *B. burgdorferi* (*s.s.*) (6%) [3]. Of our 12 locations, only Vaals shows a similarly diverse genospecies composition, which, given that Vaals is the most continentally located of our study sites in the Netherlands and possibly more similar to Luxemburg and Switzerland than the other 11 study locations, may not be surprising. A study in northern Belgium (a region bordering the Netherlands) reported a similar dominance of *B. afzelii* compared to the other genospecies [35]. In our study, *B. afzelii* was shown to be the most prevalent genospecies in the questing nymphs in the Netherlands, with higher percentages than have been reported previously for this region in meta-analyses [31, 39], and that *B. garinii*, *B. valaisiana* and *B. burgdorferi* (*s.s.*) are less prevalent but in some locations make up a substantial part of the infections, while *B. spielmanii* and *B. turdi* are only rarely detected in questing nymphs. The genospecies could not be determined for a substantial proportion of the infected nymphs, possibly due to the low *Borrelia* load in the sample or of coinfection by several *Borrelia* genospecies. While it cannot be excluded that some genospecies may be slightly harder to detect (which would have created a bias), we assume that the analysis of 60% of the infections provided us with a reasonably reliable estimate of the true genospecies distribution. Given that the genospecies composition is likely to be a result of the local composition of the host animals, we recommend that in future studies, data on host densities should be collected.

## Conclusions

Our analysis of *Borrelia burgdorferi* (*s.l.*) infection data from a longitudinal study with monthly collections of ticks in 12 locations throughout the Netherlands revealed that: (i) prevalence of infection in nymphs shows a seasonal pattern, with increasing prevalence values between March and August and a decrease afterwards; (ii) the mean prevalence of *B. burgdorferi* (*s.l.*) infections in nymphs decreased slightly between 2009 and 2016, whereas the mean density of infected nymphs did not change, as the decrease in prevalence was counteracted by an increase in the density of nymphs; (iii) variation in the density of infected nymphs was mostly caused by variation in the density of nymphs, rather than in the infection prevalence; (iv) *Borrelia afzelii* was the most commonly observed genospecies, with 75% of the infected nymphs being infected with this genospecies.

## Figures and Tables

**Fig. 4:**
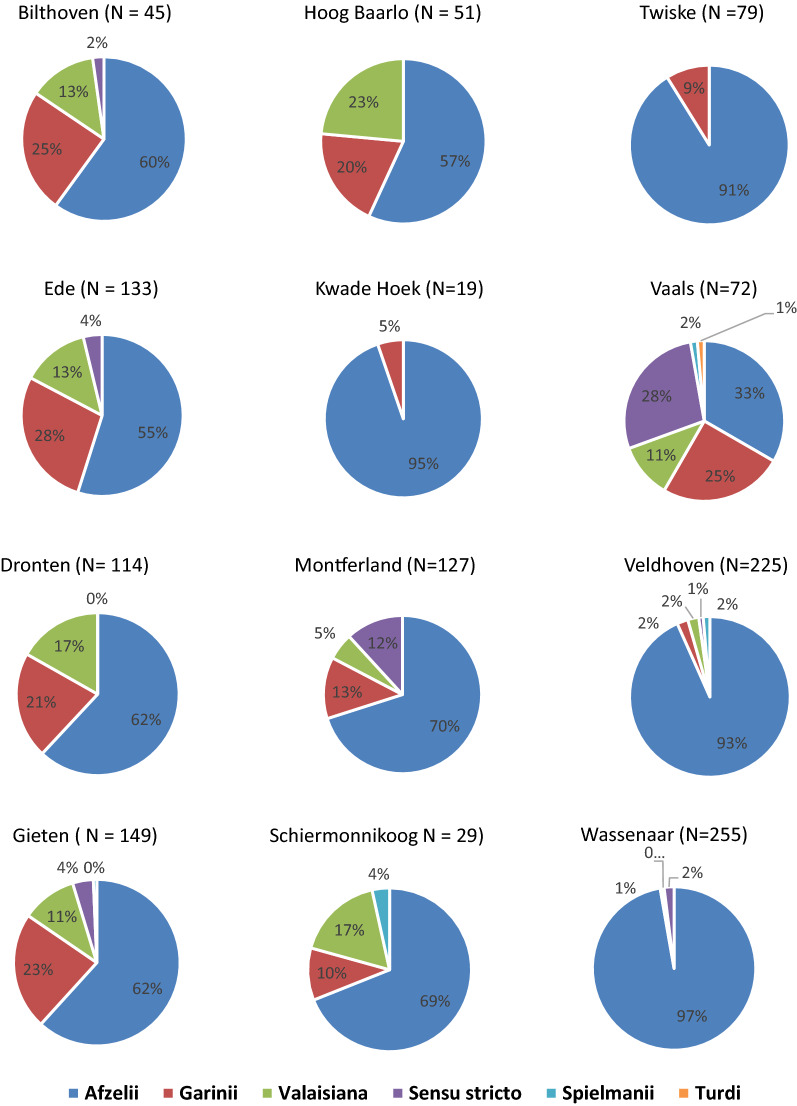
Genospecies composition per location. The percentage of infections with *B. afzelii*, *B. garinii*, *B. valaisiana*, *B. burgdorferi* (*s.s.*) and *B. spielmanii*. *B. turdi* was observed only once, in Vaals. Percentages are based on infected nymphs for which the genospecies could be determined

## Data Availability

The datasets during and/or analysed during the current study available from the corresponding author on reasonable request.

## Appendix A

See Table 2.

**Table 2:** Description of the 100-m transects where ticks were collected

Transect (coordinates)	Location coordinates	Vegetation type and dominant tree species
Bilthoven 1	52°09′22′′N, 5°13′40E	Betulo Quercetum roboris deschampsietosum (*Q. robur)*
Bilthoven 2	52°09′27′′N, 5°13′47″E	Leuco-Pinetum deschampsietosum (*P. sylvestris)*
Ede 1	52°01′41′′N, 5°41′49″E	Leucobryo-pinetum vaccinietosum (*P. sylvestris, B. pendula, Larix sp.)*
Ede 2	52°01′41′′N, 5°41′49″E	Betulo - Quercetum roboris vaccinietosum (*Q. robur)*
Dronten 3	52°27′01′′N, 5°46′25″E	Salicetum cinereae salitosum repentis (*S. caprea, Q. robur*)
Dronten 4	52°36′17′′N, 5°38′20″E	Violo odoratae-ulmetum inops (*F. sylvaticus, Q. robur. Alnus glutinosa, Acer pseudoplatantus, Fraxinus sp.*)
Gieten 1	53°00′54′′N, 6°45′18″E	Betulo Quercetum roboris deschampsietosum (*B. pubescens, Popules tremula*
Gieten 2	53°00′56′′N, 6°45′12″E	Fago Quercetum holcetosum (*Q. robur, B. pubescens, Corylus avellane*)
Hoog Baarlo 1	52°06′20′′N, 5°52′29″E	Betulo Quercetum roboris vaccinietosum *(Q. robur, Q. petrea, P. sylvestris, B. pendula)*
Hoog Baarlo 2	52°06′20′′N, 5°52′29″E	Betulo Quercetum roboris vaccinietosum *(Q. robur, Q. petrea, P. sylvestris, B. pendula)*
Kwade Hoek 1	51°50′25′′N, 3°58′58″E	Hippophao-sambucetum (*Sambucus nigra. Hippophae rhamnoides*)
Kwade Hoek 2	51°50′25′′N, 3°58′58″E	Rhamno Crataegetum (Hippophae rhamnoides)
Montferland 1	51°53′29′′N, 6°13′10″E	Leucobryo-pinetum vaccinietosum *(B. pendula, Q. robur, Picea abies, Larix sp.)*
Montferland 2	51°55′42′′N, 6°13′23″E	Quercetea Robori-Petraeae [Robinianum] *(Robinia pseudoacacia, Q. robur)*
Schiermonnikoog 1	53°29′34′′N, 6°09′46″E	Covallario Quercetum dunense (Westhof & Den Held 1969) *(B. pendula, B. pubescens)*
Schiermonnikoog 2	53°29′44′′N, 6°09′55″E	Carex arenaria-Calamogrostis epigejos-[Dicrano-Pinion] *(P. nigra)*
Twiske 1	52°26′52′′N, 4°53′35″E	Artemisio-salicetum albae agrostietosum stoloniferae *(S.alba, S. caprea)*
Twiske 2	52°26′52′′N, 4°53′35″E	Lychnido-Hypericetum Tetrapteri orchietosum morionis *(Hardly any trees: Salix alba, Salix caprea )*
Vaals 1	50°47′38′′N, 5°57′19″E	Stellario-carpinetum allietosum *(Prunus, Ulmus, Q. robur, Acer)*
Vaals 2	50°45′48′′N, 5°58′57″E	Luzulo Luzuloides Fagetum
Veldhoven 1	51°25′19′′N, 5°20′02″E	Betulo Quercetum roboris molinietosum *(Q. robur, P. sylvestris)*
Veldhoven 2	51°25′19′′N, 5°20′02″E	Betulo Quercetum roboris molinietosum *(Q. robur, P. sylvestris)*
Wassenaar 1	52°09′32′′N, 4°21′41″E	Carex arenaria - [Dicrano - Pinion] *(P. nigra)*
Wassenaar 3	52°09′34′′N, 4°21′33″E	Calamagrostis epigejos - [Dicrano - Pinion] *(P. nigra)*

*Q.*,* Quercus*;* B.*,* Betulus*;* P.*,* Pinus*;* S.** Salix*

## Appendix B

See Table 3.

**Table 3:** Total DON, NIP and DIN per year and location (over 200 m^2^ and multiple sessions)

Location	Year	2009	2010	2011	2012	2013	2014	2015	2016	Total
Bilthoven	DON	46	64	129	45	156	46	162	73	721
	NIP	0.26	0.18	0.15	0.05	0.08	0.02	0.08	0.15	0.12
	DIN	12	12	20	2	12	1	13	11	83
Ede	DON	551	315	230	190	320	208	270	215	2,289
	NIP	0.23	0.13	0.16	0.20	0.15	0.15	0.07	0.05	0.14
	DIN	127	40	36	37	49	32	18	10	318
Dronten	DON	99	241	97	62	187	84	48	15	833
	NIP	0.28	0.34	0.45	0.16	0.23	0.23	0.31	0.20	0.29
	DIN	28	82	44	10	44	19	15	3	244
Gieten	DON	334	327	1116	739	908	445	723	179	4771
	NIP	0.20	0.13	0.10	0.07	0.16	0.14	0.10	0.12	0.13
	DIN	66	43	117	55	149	62	71	22	597
Hoog Baarlo	DON	190	217	236	365	622	276	177	275	2,358
	NIP	0.13	0.09	0.09	0.05	0.02	0.03	0.08	0.02	0.06
	DIN	24	20	22	19	9	9	14	5	142
Kwade Hoek	DON	18	68	6	12	17	8	47	0	176
	NIP	0.11	0.25	0.33	0.10	0.25	0.00	0.38	NA	0.25
	DIN	2	17	2	1	4	0	18	NA	43
Montferland	DON	385	411	282	132	485	185	535	256	2,671
	NIP	0.13	0.19	0.19	0.15	0.21	0.10	0.10	0.08	0.14
	DIN	49	76	55	19	102	19	51	20	382
Schiermonnikoog	DON	26	60	47	57	42	27	39	24	322
	NIP	0.17	0.12	0.21	0.13	0.07	0.30	0.18	0.08	0.15
	DIN	4	7	10	7	3	8	7	2	49
Twiske	DON	224	314	207	227	199	253	302	431	2,157
	NIP	0.22	0.17	0.20	0.14	0.08	0.10	0.04	0.12	0.13
	DIN	49	52	41	32	16	25	12	51	272
Vaals	DON	144	131	211	105	120	152	200	111	1,174
	NIP	0.13	0.11	0.06	0.10	0.17	0.08	0.16	0.11	0.11
	DIN	18	15	12	11	21	12	32	12	134
Veldhoven	DON	237	384	660	510	776	493	1144	386	4,590
	NIP	0.14	0.17	0.28	0.19	0.11	0.07	0.14	0.24	0.16
	DIN	33	64	183	95	84	37	160	93	741
Wassenaar	DON	353	499	567	541	576	649	1173	227	4,585
	NIP	0.18	0.23	0.25	0.24	0.21	0.17	0.17	0.20	0.20
	DIN	63	117	140	128	121	107	198	45	910
All Locations	DON	2,607	3,031	3,788	2,987	4,408	2,826	4,820	2,191	2,6658
	NIP	0.18	0.18	0.19	0.14	0.14	0.12	0.12	0.12	0.15
	DIN	464	537	714	421	637	327	584	272	3,925

Total number of nymphs (DON), prevalence (NIP) and estimated number of infected nymphs (DIN) per year and location (over 200 m^2^ and in multiple sessions). Prevalence (NIP) is calculated for each year–location combination by dividing the total number of* Borrelia*-positive nymphs by the total number of tested nymphs. For Vaals, the DIN and DON are based on a single transect from 2014 onwards because the other transect was not sampled after December 2013

## Appendix C

See Table 4.

**Table 4:** Total DON, NIP and DIN per 200 m^2^, per month and per location

Location	Variable	January	February	March	April	May	June	July	August	September	October	November	December
Bilthoven	DON	0	3	8	57	80	184	113	127	59	64	24	2
	NIP	NA	0.00	0.13	0.07	0.14	0.13	0.12	0.13	0.14	0.08	0.04	0.00
	DIN	NA	0	1	4	11	23	13	17	8	5	1	0
Dronten	DON	0	0	5	112	126	143	118	94	97	89	49	0
	NIP	NA	NA	0.00	0.30	0.19	0.33	0.26	0.31	0.36	0.34	0.29	0.00
	DIN	NA	NA	0	33	23	47	31	29	35	31	14	0
Ede	DON	9	11	83	351	476	549	220	262	165	111	51	12
	NIP	0.22	0.00	0.09	0.13	0.11	0.14	0.12	0.18	0.17	0.18	0.10	0.09
	DIN	2	0	7	44	55	78	27	48	28	19	5	1
Gieten	DON	10	3	60	253	418	965	1039	828	688	406	91	10
	NIP	0.00	0.33	0.10	0.10	0.12	0.13	0.19	0.12	0.12	0.13	0.08	0.10
	DIN	0	1	6	25	52	124	197	96	81	51	7	1
Hoog Baarlo	DON	7	3	23	281	475	597	264	347	205	97	55	4
	NIP	0.00	0.00	0.07	0.04	0.07	0.06	0.06	0.09	0.04	0.03	0.09	0.00
	DIN	0	0	2	12	33	35	17	32	8	3	5	0
Kwade Hoek	DON	0	0	0	14	80	56	11	4	0	5	6	0
	NIP	NA	NA	NA	0.21	0.27	0.30	0.18	0.00	NA	0.00	0.00	NA
	DIN	NA	NA	NA	3	21	17	2	0	NA	0	0	NA
Montferland	DON	2	15	56	446	478	670	414	224	142	139	76	9
	NIP	0.00	0.07	0.15	0.14	0.11	0.15	0.17	0.18	0.14	0.12	0.14	0.00
	DIN	0	1	8	62	54	102	72	41	20	16	11	0
Schiermonnikoog	DON	1	3	7	41	53	91	56	24	15	18	7	6
	NIP	0.00	0.00	0.00	0.15	0.19	0.15	0.16	0.13	0.13	0.22	0.00	0.20
	DIN	0	0	0	6	10	13	9	3	2	4	0	1
Twiske	DON	10	8	34	344	602	831	112	27	43	61	77	8
	NIP	NA	0.25	0.18	0.10	0.11	0.13	0.14	0.09	0.12	0.15	0.19	0.13
	DIN	NA	2	6	34	67	107	16	3	5	9	14	1
Vaals	DON	1	3	17	81	124	121	120	101	67	47	24	3
	NIP	0.00	0.00	0.12	0.03	0.12	0.08	0.14	0.17	0.08	0.16	0.04	0.33
	DIN	0	0	2	2	15	10	16	18	5	7	1	1
Veldhoven	DON	26	56	230	890	1171	859	419	247	294	213	169	16
	NIP	0.27	0.21	0.11	0.15	0.15	0.18	0.17	0.13	0.17	0.17	0.19	0.12
	DIN	7	12	26	135	179	158	70	32	50	36	33	2
Wassenaar	DON	12	10	119	841	844	823	576	410	356	408	162	24
	NIP	0.33	0.33	0.08	0.17	0.15	0.24	0.18	0.26	0.27	0.19	0.17	0.12
	DIN	4	3	10	141	130	201	105	105	98	79	28	3
All Locations	DON	78	115	642	3711	4927	5889	3462	2695	2131	1658	791	94
	NIP	0.19	0.16	0.11	0.13	0.13	0.16	0.16	0.17	0.16	0.16	0.15	0.11
	DIN	13	19	68	503	650	916	576	424	340	261	119	10

Total number of nymphs (DON), average prevalence (NIP) and estimated number of infected nymphs (DIN) per month and location (over 200 m and over 7.5 years). Prevalence (NIP) is calculated for each month–location combination by dividing the total number of infected nymphs captured in a certain month (over several years) by the total number of tested nymphs. DON is the sum of all nymphs captured in that month (over 7.5 years) and DIN is the (rounded-off) product of DON × NIP

## Appendix D

See Fig. 4.

## Appendix E

See Table 5.

**Table 5:** Results trend over time (years) for each location

Location	NIP^a^			DON^b^			DIN^b^		
	Estimate	Odds ratio	*p*	Estimate	Ratio	*p*	Estimate	Ratio	*p*
	β	e ^β^							
Bilthoven	*− 0.1949*	*0.823*	*0.0037*	0.1669	1.1816	0.0071	*− 0.0473*	0.9538	*0.4939*
Dronten	*− 0.0700*	*0.932*	*0.3200*	-0.1908	0.8263	0.0180	− 0.2273	0.7967	0.0100
Ede	− 0.1787	0.836	0.0014	0.0152	1.0153	0.7629	− 0.224	0.7993	7.68e-06
Gieten	− 0.0453	0.956	0.3890	0.1024	1.1078	0.0729	0.0493	1.0505	0.433
Hoog Baarlo	− 0.2172	0.805	0.0035	0.1622	1.1761	0.0037	*− 0.1464*	0.8638	*0.0185*
Kwade Hoek	0.1292	1.030	0.1316	*− 0.0101*	0.9900	0.9357	*0.1211*	1.1287	*0.5589*
Montferland	− 0.1024	0.903	0.0458	0.0365	1.0372	0.3709	*− 0.0799*	0.9232	*0.218*
Schiermonnikoog	*0.0109*	*1.011*	*0.8920*	0.0004	1.0004	0.9928	*− 0.0631*	0.9388	*0.4740*
Twiske	− 0.1877	0.829	0.0003	0.0966	1.1014	0.0359	− 0.0926	0.9116	0.2075
Vaals	NA^c^	NA^c^	NA^c^	− 0.0866	0.9170	0.2849	*0.0327*	1.0332	*0.7296*
Veldhoven	− 0.0074	0.993	0.9256	0.2385	1.2693	5.72e-08	0.1069	1.1128	0.2268
Wassenaar	− 0.0547	0.947	0.0419	0.0959	1.1006	0.0241	0.0747	1.0776	0.0926
All locations	− 0.0843	0.919	4.04e-05	0.0705	1.0730	0.0011	− 0.0414	0.9594	0.1130

Estimated effect of the numerical value “Year” in model, which indicates the trend over time (over years)

Model outcomes in italics indicate that the model had convergence problems, and we used the squared absolute difference between the month and June (M = (|6-Month|)^2^ instead of Month

^a^For the NIP, the estimate β is obtained using the beta-binomial model, with a logit link. The corresponding estimated odds ratio (calculated as e^β^), indicates the ratio between the odds of a tick being infected in 1 year and the next, with an odds ratio > 1 indicating an increase in the prevalence over time and an odds ratio < 1 indicating a decrease

^b^ For the DON and DIN (estimated from negative binomial models with log link), the estimated β can be used to calculate the ratio (e^β^) of the estimated numbers of ticks between two consecutive years, with an odds ratio > 1 indicating an increase in numbers and an odds ratio < 1 indicating a decrease in the numbers

^c^NA indicates that the model-fitting algorithm did not converge

## Appendix F

See Table 6.

**Table 6:** Pairwise comparisons of the estimated marginal NIP values between locations

Forest type	Location	Bilthoven	Ede	Gieten	Hoog Baarlo	Montferland	Veldhoven	Kwade Hoek	Schiermonnikoog	Wassenaar	Twiske	Vaals	Dronten
Poor mixed forest	Bilthoven	x											
Rich mixed forest	Ede		x										
	Gieten			x									
	Hoog Baarlo		**	**	x								
	Montferland				**	x							
	Veldhoven				***		x						
Dunes/coast	Kwade Hoek				***			x					
	Schiermonnikoog				**				x				
	Wassenaar	*		*	***					x			
Marshland	Twiske				**						x		
Oak, hornbeam and beech dominated	Vaals				*					**		x	
Willow and elm woodland	Dronten	***	**	**	***	**	*				**	***	x

Results of the pairwise comparison of the estimated marginal means for NIP between the locations

**p* < 0.05, ***p* < 0.01, ****p* < 0.0001
